# Iran COVID-19 Epidemiology Committee: A Review of Missions, Structures, Achievements, and Challenges

**DOI:** 10.34172/jrhs.2021.45

**Published:** 2021-03-07

**Authors:** Shahin Amiri, Aliakbar Haghdoost, Ehsan Mostafavi, Hamid Sharifi, Niloofar Peykari, Ahmad Raeisi, Mohammad Assai Ardakani, Mohsen Asadi Lari, Hamid Soori, Afshin Ostovar, Babak Eshrati, Mohammad Mehdi Gouya, Mahshid Nasehi, Seyed Mehdi Tabatabaei, Manzar Amirkhani, Sana Eybpoosh

**Affiliations:** ^1^Biotechnology Research Center, Medical Biotechnology Department, Pasteur Institute of Iran, Tehran, Iran; ^2^Student Research Committee, Pasteur Institute of Iran, Tehran, Iran; ^3^HIV/STI Surveillance Research Center, and WHO Collaborating Center for HIV Surveillance, Institute for Futures Studies in Health, Kerman University of Medical Sciences, Kerman, Iran; ^4^Department of Epidemiology and Biostatistics, Research Centre for Emerging and Reemerging Infectious Diseases, Pasteur Institute of Iran, Tehran, Iran; ^5^Development of Research and Technology Center, Deputy of Research and Technology, Ministry of Health and Medical Education, Tehran, Iran; ^6^Department of Medical Entomology and Vector Control, School of Public Health, Tehran University of Medical Sciences, Tehran, Iran; ^7^Malaria Control Unit, Center for Communicable Diseases Control, Ministry of Health and Medical Education, Tehran, Iran; ^8^Ministry of Health and Medical Education, Tehran, Iran; ^9^Department of Epidemiology, School of Public Health, Iran University of Medical Sciences, Tehran, Iran; ^10^Oncopathology Research Center, Iran University of Medical Sciences, Tehran, Iran; ^11^Safety Promotion and Injury Prevention Research Center, Shahid Beheshti University of Medical Sciences, Tehran, Iran; ^12^Osteoporosis Research Center, Endocrinology and Metabolism Clinical Sciences Institute, Tehran University of Medical Sciences, Tehran, Iran; ^13^Preventive Medicine and Public Health Research Center, Iran University of Medical Sciences, Tehran, Iran; ^14^Center for Control of Communicable Diseases, Ministry of Health and Medical Education, Tehran, Iran; ^15^Health Promotion Research Center, Zahedan University of Medical Sciences, Zahedan, Iran; ^16^National Committee of COVID-19 Epidemiology, Deputy of Education, Ministry of Health and Medical Education, Tehran, Iran

**Keywords:** Committee Membership, COVID-19, Epidemiology, Iran, Pandemics

## Abstract

**Background:** Since the beginning of the coronavirus disease 2019 (COVID-19) epidemic in Iran, the control and management of the epidemic were headed by the National Headquarter for the Control of COVID-19 Epidemic through setting up different scientific committees, including the COVID-19 National Epidemiology Committee. The present study reviews the missions, structures, achievements, and challenges of the Epidemiology Committee.

**Study design:** A rapid review

**Methods:** All relevant reports, documents, guidelines, published literature, and surveillance data related to the establishment, visions, missions, roles, activities, and outputs of the COVID-19 Epidemiology Committee were critically reviewed in this study.

**Results:** The efforts of the committee’s working groups may have impacted improvements in data registration/usage, provincial data quality at provincial levels, and perception of the epidemic situation in the provinces. The committees have also played role in informing the policies in different stages of the epidemic through routine or problem-based data/evidence analyses, epidemic investigations, and mathematical modeling.

**Conclusions:** The structure and experience gained by the committee can be used in similar situations within and outside the country. To further improve the impacts of our activities, it is essential to have effective interaction, collaboration, and data flow between the committee and a broad range of organizations within and outside the Ministry of Health and Medical Education.

## Introduction

Since the beginning of 2020, the coronavirus disease 2019 (COVID-19) pandemic has affected more than 200 countries worldwide, with millions of infections and thousands of mortalities ^1,2^. Iran was among the first countries infected with the virus after the epidemic in Wuhan, China ^3^. The primary cases in Iran were identified and reported on 19 February 2020 in Qom ^4^. Soon after that, many more cases were identified in other provinces of the country, with some provinces being highly affected in the early phase of the epidemic, including Tehran, Gilan, Mazandaran, Qom, and Golestan ^5^.

Since the start of the epidemic, Iran has adopted a wide range of control policies in order to mitigate the rate of epidemic spread. One of the key efforts involved the establishment of the National Headquarter for the Control of COVID-19 Epidemic (NHCCE). The Supreme National Security Council assigned headquarters as the principal entity responsible for the development and communication of COVID-19 mitigation policies. The council appointed Iran’s minister of health as the leader of the NHCCE ^6,7^.

 After the establishment of the NHCCE, different scientific committees were formed by the NHCCE leader in order to provide scientific support for mitigation policies in each area. The COVID-19 National Epidemiology Committee, as one of these scientific committees, was formed in February 2020 in the Ministry of Health and Medical Education (MoHME). This study reviews the missions, objectives, structures, achievements, and challenges of the National COVID-19 Epidemiology Committee in Iran by October 2020.

Learning from experiences has been widely recommended by leadership scholars ^8,9^. It has been discussed that countries that have had and used their previous experiences in dealing with emerging infections, such as China and South Korea, have been better able to efficiently respond to the COVID-19 pandemic ^10^. Notably, some countries without previous experience also acted successfully in responding to the epidemic. The experience of these countries is of high value and needs to be disseminated in more detail. Some of the known lessons from New Zealand’s pandemic response include rapid science-based risk assessment linked to early decisive government action, implementation of interventions at various levels (e.g., border-control measures, community-transmission control measures, and case-based control measures), provision of empathic leadership, and effective communication of key messages to the public by the prime minister ^11^. For the experiences to be re-usable, it is necessary to have them published. In many countries, different health sectors/committees/disciplines have initiated publishing their experiences and lessons learned from responding to the COVID-19 pandemic ^12-19^.

 This review aimed to convey the experience gained by the COVID-19 Epidemiology Committee in Iran, including the lessons learned by all its achievements and shortcomings/barriers, to help leaders better organize similar activities in the future. The mission is to compile the results of local and international epidemiological studies, mathematical models, and expert opinions in order to promote evidence-oriented policymaking. To achieve this goal, the committee has the following objectives:

- The investigation of the current status and prediction of the trend of COVID-19 in the country
- The investigation of the epidemiological models of COVID-19 in the country and the world
- Leading the epidemic investigations of COVID-19 at national and subnational levels
- A review of the documents and findings of studies published in high-standard scientific journals about COVID-19

## Methods

 The current study critically reviewed all relevant reports, documents, guidelines, published literature, and surveillance data related to the establishment, visions, missions, roles, activities, and outputs of the COVID-19 Epidemiology Committee in Iran.

## Results

###  Structures 

 A. Intrasectoral collaborators

 A diverse group of multidisciplinary intrasectoral collaborators is deemed necessary to accomplish the missions of the committee. Therefore, the committee collaborates with the following entities as its major intrasectoral collaborators:

- The NHCCE
- Vice-Chancellor of Health Affairs, MoHME, Iran
- Vice-Chancellor in Treatment Affairs, MoHME, Iran
- Deputy of Research and Technology, MoHME, Iran
- Food and Drug Organization, MoHME, Iran
- Deputy of Nursing, MoHME, Iran
- Statistics and Information Technology Center, MoHME, Iran
- National Diagnostic Laboratory Network, Pasteur Institute of Iran
- Medical Sciences universities

B. Intersectoral collaborators

- World Health Organization Country Office, Iran
- Iranian Academy of Medical Sciences, Iran
- Research Center of the Islamic Consultative Assembly, Iran
- Sharif University of Technology, Iran

###  Members and colleagues

 In order to achieve its missions, a wide range of expertise formed the core members of the COVID-19 Epidemiology Committee. The composition brings together scientific and/or executive managers/members in various health-related fields, either in the MoHME or medical universities. This has provided a more comprehensive perspective of the epidemic features. Such diversity has also facilitated the intersectoral communication and information flow with the areas at the disposal of committee members. In addition to its core members, the committee has a close working relationship with more than 100 colleagues. These colleagues mainly collaborate within one or more working groups of the committee.

###  Working groups

**Figure 1:**
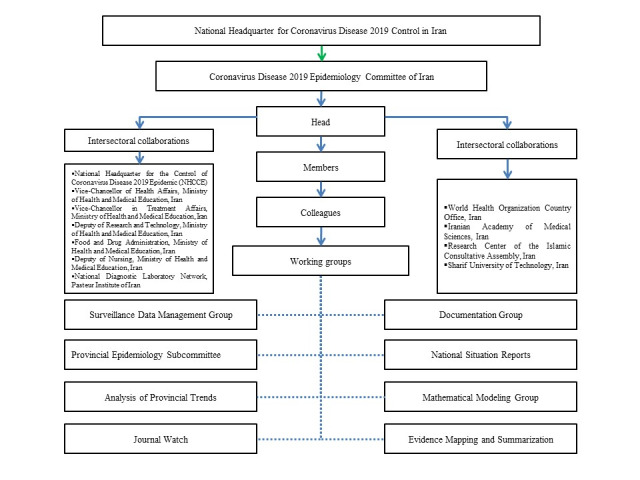
Organizational chart of the National Coronavirus Disease 2019 Epidemiology Committee in Iran
 A. COVID-19 Surveillance Data Management Core Group

A diverse set of working groups was formed in order to aid the committee to achieve its objectives. The description of each working group and its main tasks, outputs, and achievements are provided below. Figure 1 also illustrates a schematic representation of the organizational chart of the COVID-19 Epidemiology Committee and the list of its working groups.

*Description and objectives*: The objectives of the COVID-19 Surveillance Data Management Core Group include compiling, organizing, cleaning, and analyzing COVID-19 surveillance data. The National COVID-19 data are deposited in some independent databases (including Medical Care Monitoring Center [MCMC] database and Hospital Information System [HIS]), which contain the clinical data of hospitalized suspected and confirmed COVID-19 cases, and SIB portal, which contains outpatients’ data and screening information. The working group manages data cleaning and analysis, the main results of which are communicated with policymakers to inform decision processes. The working group also uses the data to monitor the accuracy of surveillance data collection, registration, and flow from the local settings (i.e., hospitals and medical universities) to upper levels within the MoHME. Feedback is provided to the working entities whenever needed.

*Outputs and impacts*: The work performed in this group has improved data registration of hospitals and medical universities. The results of these analyses have also informed the preliminary case definition and clinical management guidelines for COVID-19 in Iran and many decisions processed to control the epidemic in the country.

 B. Provincial Epidemiology Subcommittee

*Description and objectives*: This working group was formed to facilitate the provision of high-resolution perspectives on the epidemiological features of COVID-19 within each province. To this end, provincial COVID-19 epidemiology committees were formed in each province. The committees were responsible for leading province-level outbreak investigation, contact tracing, epidemiological data gathering, and surveillance data cleaning and analysis. The committees were responsible for providing weekly reports about the epidemiological profile of the province to the National Epidemiology Committee. Feedbacks were provided whenever needed. The National COVID-19 Epidemiology Committee also facilitated logistic issues faced by the provincial committees.

*Outputs and impacts*: During the early months of the epidemic, investigations were weekly prepared by provincial committees and dispatched to the COVID-19 Epidemiology Committee. Subsequently, the reports were sent once a month. These reports are now the premise of provincial determination and intervention measures.

 C. Analysis of Provincial Epidemic Trends

*Description and objectives*: This working group aimed to determine the epidemic trend of COVID-19 in different provinces of Iran. Accordingly, since the beginning of the epidemic and based on the rate of COVID-19 cumulative incidence, the provinces of Iran are classified into three categories, namely high-, medium-, and low-incidence provinces. The provinces can move between these categories over time given their epidemic status. The trend of daily confirmed cases and mortalities are analyzed every 7 days. In each analysis, the changes in the number of confirmed and mortality cases within the past 5 days are calculated by averaging the numbers in a specified day and a day before and after that. Given the trend of morbidity and mortality, the provincial trend is inferred as upward, downward, or stable. These analyses show if a province approaches a new epidemic pick or has passed one. Provinces with controversial data can also be detected in these analyses, where the source of controversy can then be identified.

*Outputs and impacts*: The factsheets published by this working group provided the policymakers at the MoHME and medical universities with information about the possible course of the epidemic in different provinces. These analyses can detect incompatibility in the data of the provinces. In such cases, the feedback was immediately provided for the province, which has ultimately improved the reporting quality of the provinces.

 D. National Daily Situation Reports for COVID-19

*Description and objectives*: The National Daily Situation Reports for COVID-19 working group was formed to provide national COVID-19 situation reports for a broad audience of caring individuals within and outside the country. The national COVID-19 surveillance data is being analyzed, validated, and aggregated. The data sources for daily reports mainly include the MCMC and HIS databases (for inpatients) as well as the SIB portal (for outpatients). The reports contain basic epidemiological features of confirmed and mortality cases, including the national daily trend of infection, mortality, and recovery, age and gender distributions, prevalence of different comorbidities, and trends of case fatality rate and intensive care unit admission.

*Outputs and impacts*: By 20 October 2020, 62 daily situation reports have been released by the National Epidemiology Committee both in Persian and English, some of which have provided basic information required for policymaking either in the clinical or public health settings (access to daily situation reports ^20-27^).

 E. Model-Based Analyses and Forecast of COVID-19 Trend

*Description and objectives*: To inform the decision, the modeling working group was responsible for the development of simulation-based analyses to respond to the questions coming up over the course of the epidemic. For this purpose, the first modeling report forecasted the possible number of COVID-19 infections, hospitalizations, and mortalities in the whole country and Tehran (i.e., the capital of Iran) using compartmental dynamic models. The first modeling report was the estimation of the number of medical equipment required under different levels of social distancing. The results were used to inform policies and practices at the moment. The models were developed based on a domestic model in collaboration with Modeling in Health Research Center, Institute for Futures Studies in Health, Kerman University of Medical Sciences, Kerman, Iran, and using an international developed model (i.e., COVID-19 International Modelling [CoMo model]) in collaboration with the University of Oxford, England. Other models that are developed upon emerging practical questions include the prediction of the effects of closing schools and universities, holy shrines, and other nonpharmaceutical interventions on the number of COVID-19 infections, hospitalizations, and mortalities. Moreover, some reports on the burden of disease and economic impact of the epidemic were prepared (access to daily situation reports ^20-27^).

*Outputs and impacts*: Eight policy reports (seven based on domestic models and one based on the CoMo model) were delivered to the MoHME, evaluating the effect of interventions or predicting future trends.

 F. Evidence Mapping and Summarization

*Description and objectives*: The mission of this working group is the improvement of the current research awareness by providing the audiences with a summary report of the high-quality evidence in Persian. Major activities include the provision of summary research and journal watches in Persian. To this end, scientific information and research results of COVID-19 from various international and domestic credible sources, including scientific journals, related organizations, news agencies, and research centers, are collected by a group of 20 students and health experts every day. The topics cover various aspects of COVID-19. The summarization task is performed using a standard “data extraction” template. The template contains different headings that should be filled, including “main results”, “key message of the study”, “study title”, “article publication date”, “journal name”, “target audiences”, “study samples”, “conclusions”, and “lessons to be learned”. These summaries are utilized by a wide range of audiences, including policymakers, managers, therapists, and the general population.

**Figure 2:**
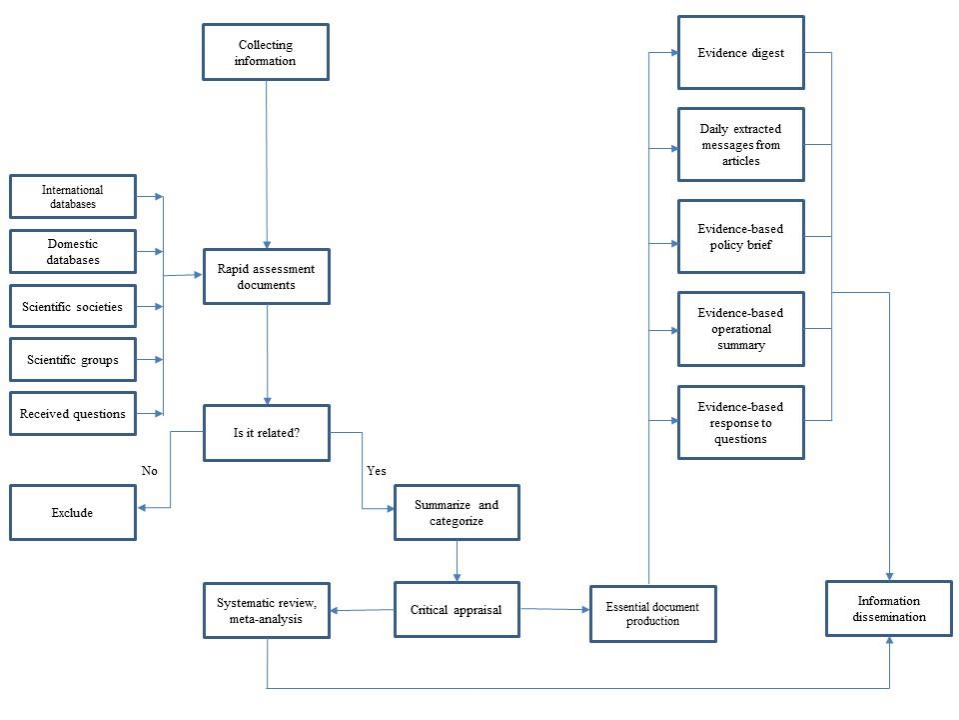
Knowledge translation and dissemination pathway in the Coronavirus Disease 2019 Epidemiology Committee in Iran

Similar to the reports of other working groups, the above-mentioned documents are also uploaded regularly on the official website of the COVID-19 Epidemiology Committee. These materials are also distributed within a broad network of audiences either in hard copy or electronic formats. Figure 2 depicts the knowledge translation and dissemination pathway followed in the COVID-19 Epidemiology Committee.

*Outputs and impacts*: Within the 4 months, more than 850 evidence summaries have been uploaded, receiving more than 200,000 website views by the target audiences. The process takes about 2-3 h every day.

 G. Documentation Core Group

*Description and objectives*: The objectives of this working group were to share Iran experiences for the control of the COVID-19 pandemic that can be used in future emergencies, document efforts/ interventions, success stories, challenges, innovations, and suggestions for the better management of emergencies by the chancellor of universities, all relevant sectors, and senior management within MoHME. In addition to its core members, this working group enjoyed the collaboration and engagement of a focal point from each of the universities of medical sciences and health services and a focal point from 28 relevant departments within MoHME. This working group interviewed around 30 heads of the organization and deputy ministers from other sectors that were documented in one of the below-published documents.

*Outputs and impacts*: To date, four documents have been published that are summarized below. In addition, two others are on the way (i.e., one interview with 65 chancellors of universities of medical sciences and health services all over the country and the last one interview with Senior Management at the MoHME including Minister of Health and all his deputies).

 1. Islamic Republic of Iran actions in prevention, diagnosis, and management of COVID-19 that is published in 150 copies in 88 pages and shared on different websites. The document summarizes MoHME policies, strategies, and action points during the first 2 months of the pandemic in 6 main sections, namely governance, action points, international and public relations, emergency unit and Red Crescent actions, provision of medicines and personal protective equipment, and medical education, research, and students affairs.

 2. “The First-Line Care Providers” that is published in 100 copies in 70 pages. Considering substantial efforts of the care providers, namely community health workers at rural health houses, family experts at urban health posts, nurses and midwives at hospitals, and emergency and laboratory experts, it was decided to document some examples of dedicated and sacrificed efforts of these first-line care providers in the current review.

 3. Intersectoral Collaboration and Partnership is published in 200 copies in 164 pages and shared on different websites. In this study, the researchers identified a few relevant questions related to each sector and made an appointment with the head of the organization/deputy ministers in different sectors. The members of the documentation committee were divided into three main groups and met the highest authority within the relevant sectors. The main interventions, challenges, innovations, and suggestions for improvement in emergency management were also among the key questions raised in these interviews.

 4. Preparedness and Response for the Control of COVID-19 (in English) in Islamic Republic of Iran that is published in 200 copies in 82 pages and shared on different websites in addition to shared hard copies to all United Nation agencies and foreign embassies located in Tehran. To share the policies, strategies and main interventions of Iran in controlling the COVID-19 pandemic and in line with Global World Health Organization guidelines for emergency preparedness and response, this document is prepared in partnership and coordination with the relevant departments within MoHME. The main contents of the document include epidemiological studies, health governance, policies and strategies for the prevention, diagnosis, and treatment of COVID-19, screening standards, implementation of health protocols, use of the Primary Health Care (PHC) network in national screening, community engagement and empowerment, international collaboration and partnership, summary of nursing, midwifery, and emergency units, and Red Crescent Society and environmental health protocols and standards.

Table 1 shows the main outputs of the national working groups of the COVID-19 Epidemiology Committee.

**Table 1:** Main outputs of the working groups of the National Coronavirus Disease 2019 Epidemiology Committeea^a^

**Page** **code**	**Title**	**Total** **v isits(n)**	**Creation date**	**Events (n)**	**Average** **v isits(n)**
42	Modeling	7,225	3/16/2020	14	516
60	Selected journals	17,245	3/19/2020	62	278
63	Internal articles	1,526	3/25/2020	2	763
25	Factsheets	10,893	3/07/2020	60	182
57	Public education	4,776	3/17/2020	132	36
67	Daily reports	12,450	4/07/2020	34	366
33	Proposals of scientific associations	1,636	3/07/2020	23	71
59	Guides for policymakers and managers	745	3/18/2020	11	68
64	Guides for occupations and professions	2,765	4/02/2020	45	61
65	Guides for health experts	1,426	4/03/2020	20	71
66	Clinical and therapeutic guides	1,863	4/04/2020	23	81
69	Guides for entire society	438,031	4/19/2020	12	36,503
26	Global latest studies	135,358	3/07/2020	898	151

^a^ The numbers are retrieved on October 13, 2020.

## Discussion

 The field and scope of the work in the Epidemiology Committee have a strong inter/intra-organizational character. To convert COVID-19-relevant data and information into policy, it is required to have effective communication, collaboration, and data/information flow between the committee and a wide range of sectors/organizations within and outside MoHME. To date, one of the main challenges has been effective and timely coordination between the committee and these sectors.

 The COVID-19 surveillance data are deposited in more than one database, with different databases covering part of the data, such as laboratory, outpatient, and inpatient data. Combining the aforementioned databases poses important challenges in data cleaning, data verification, and linkage of different databases. On the other hand, while putting the stress on COVID-19 data registration in the local settings has led to the completeness of the data, it has also had important side effects. Firstly, the efforts have already posed a heavy workload to the local staff, leading to occupational burnout in some staff. Secondly, emphasizing the diagnosis and data registration of COVID-19 has overshadowed the surveillance of other respiratory infections, especially influenza.

 Finally, the infection of some of the committee’s key members with COVID-19 during the early phases of the epidemic diminished the workforce that could actively collaborate with the committee to fulfill its missions. This challenge highlighted the necessity of protection against infection for those involved in critical positions during the epidemics.

## Conclusions

 The structure and the experience gained by the committee can be used in similar situations within and outside the country. To further improve the impacts of our activities, it is essential to have effective interaction, collaboration, and data flow between the committee and a broad range of organizations within and outside the MoHME.

## Acknowledgements

 We would like to thank all the colleagues in the working groups of the Epidemiology committee who contributed to the defined activities, and helped the committee to achieve its goals.

## Conflict of interest

 Aliakbar Haghdoost holds the position of the Deputy Minister of Education in MoHME and Mohammad Mehdi Gouya holds the position of the head of Iran’s Center for Disease Control and Prevention in MoHME. The rest of the authors declare that there is no conflict of interest.

## Funding

 The authors received no funding for this study.

## Highlights

- Activities: Data curing, modeling, outbreak analysis, evidence mapping, and documentation.
- The compilation of coronavirus disease 2019-related data from different sources remains a challenge.
- Rapid committee formation helped in keeping pace with the epidemic growth rate.
